# MOMC-5. Systems pharmacogenomics identifies novel targets and clinically actionable therapeutics for medulloblastoma

**DOI:** 10.1093/noajnl/vdab070.015

**Published:** 2021-07-05

**Authors:** Laura Genovesi, Amanda Millar, Elissa Tolson, Matthew Singleton, Emily Hassall, Marija Kojic, Caterina Brighi, Emily Girald, Clara Andradas, Mani Kuchibhotla, Raelene Endersby, Nicholas Gottardo, Anne Bernard, Christelle Adolphe, James Olson, Melissa Davis, Brandon Wainwright

**Affiliations:** 1 The University of Queensland Diamantina Institute, Woolloongabba, QLD, Australia; 2 Australian Institute for Bioengineering and Nanotechnology, St Lucia, QLD, Australia; 3 Fred Hutchinson Cancer Research Center, Seattle, Washington, DC, USA; 4 Telethon Kids Institute, Nedlands, WA, Australia; 5 QFAB Bioinformatics, Institute for Molecular Bioscience, St Lucia, QLD, Australia; 6 The Walter and Eliza Hall Institute of Medical Research, Parkville, VIC, USA; 7 The University of Queensland Diamantina Institute, Woolloongabba, QLD, USA

## Abstract

**Background:**

Medulloblastoma (MB) is the most common malignant paediatric brain tumour and a leading cause of cancer-related mortality and morbidity. Existing treatment protocols are aggressive in nature resulting in significant neurological, intellectual and physical disabilities for the children undergoing treatment. Clearly, there is an urgent need for improved, targeted therapies that minimize these harmful side effects.

**Methods:**

We identified candidate drugs for MB using a network-based systems-pharmacogenomics approach: based on results from a functional genomics screen, we identified a network of interactions implicated in human MB growth regulation. We then integrated drugs and their known mechanisms of action, along with gene expression data from a large collection of medulloblastoma patients to identify drugs with potential to treat MB.

**Results:**

Our analyses identified drugs targeting CDK4, CDK6, and AURKA as strong candidates for MB; all of these genes are well validated as drug targets in other tumour types. We also identified non-WNT MB as a novel indication for drugs targeting TUBB, CAD, SNRPA, SLC1A5, PTPRS, P4HB and CHEK2. Based upon these analyses we subsequently demonstrated that one of these drugs, the new microtubule stabilizing agent, ixabepilone, blocked tumour growth *in vivo* in mice bearing Sonic Hedgehog and Group 3 patient-derived xenograft tumours, providing the first demonstration of its efficacy in MB.

**Conclusions:**

Our findings confirm that this data-driven systems pharmacogenomics strategy is a powerful approach for the discovery and validation of novel therapeutic candidates relevant to MB treatment, and along with data validating ixabepilone in PDX models of the two most aggressive subtypes of medulloblastoma, we present the network analysis framework as a resource for the field.

